# Fate of Fecal Indicators in Resource-Oriented Sanitation Systems Using Nitrifying Bio-Treatment

**DOI:** 10.3390/ijerph15010164

**Published:** 2018-01-20

**Authors:** Shervin Hashemi, Mooyoung Han, Eun Namkung

**Affiliations:** 1 Department of Civil and Environmental Engineering, Seoul National University, 1 Gwanak-ro, Gwanak-gu, Seoul 08826, Korea; shervincee@snu.ac.kr; 2 Institute of Construction and Environmental Engineering, Seoul National University, 1 Gwanak-ro, Gwanak-gu, Seoul 08826, Korea; enamkung@snu.ac.kr

**Keywords:** *Escherichia coli*, *Nitrobacter winogradskyi*, *Nitrosomonas europaea*, resource-oriented sanitation, source-separated feces, total coliforms

## Abstract

Hygienic fecal treatment in resource-oriented sanitation (ROS) systems is an important concern. Although the addition of nitrifying microorganisms is a sustainable fecal treatment method in ROS systems, it is essential to examine the cleanliness of this method. In this study, we investigated the fate of fecal indicators in source-separated fecal samples through tracking *Escherichia coli* and total coliforms. The effects of adding different amounts of *Nitrosomonas europaea* bio-seed, along with a constant amount of *Nitrobacter winogradskyi* bio-seed, were studied. In intact feces samples, the pathogen population underwent an initial increase, followed by a slight decrease, and eventually became constant. Although the addition of nitrifying microorganisms initially enhanced the pathogen growth rate, it caused the reduction process to become more efficient in the long-term. In addition to a constant concentration of 10,000 cells of *N. winogradskyi* per 1 g feces, a minimum amount of 3000 and 7000 cells of *N. europaea* per 1 g feces could completely remove *E. coli* and total coliforms, respectively, in less than 25 days. Increasing the amount of bio-seeds added can further reduce the time required for total pathogen removal.

## 1. Introduction

Lack of access to sustainable sanitation is a result of several technical, economic, and social challenges of the current sanitation systems, and can lead to improper sanitation practices (i.e., open defecation), especially in low-income countries. Consequently, the sixth goal of the Sustainable Development Goals (SDG-6) aims to end open defecation by 2030 [1,2,3,4].

Resource-oriented sanitation (ROS) systems are a sustainable solution for sanitation challenges [1,5]. Such systems are based on source-separation of urine and feces and treating them onsite to be utilized as fertilizer [1,6].

Although ROS systems are effective in controlling open defecation, there are concerns about their hygienic conditions, especially in the case of source-separated feces [7]. Feces naturally contains pathogens [8]. In this case, it is essential to have a hygienic treatment process for source-separated feces so that it may be utilized as fertilizer.

Several studies examined different approaches for pathogen removal in ROS systems [9]. Magri et al. suggested the application of desiccation and urea treatment in Urine-Diverting Dry Toilet (UDDT) systems for removing pathogens in source-separated feces [10]. Composting is also a common practice, as it is useful in killing fecal pathogens, while also successfully providing a soil conditioner [11,12]. Wastewater stabilization pond (WSP) is also an economic systems that is effective in removal of fecal indicators and pathogenic bacteria [13]. Other approaches, such as temperature and pH adjustment, are effective in the reduction of harmful bacteria [14,15]. However, challenges persist in the stability and sustainability of the aforementioned methods for ROS systems, especially when there is a high amount of feces as input [16].

A suitable approach for onsite fecal treatment is required to meet the basic requirements of ROS systems. Utilizing nitrifying microorganisms not only can be useful in enhancing the degradation of source-separated feces, but also adds a specific concentration of *Nitrosomonas europaea* and *Nitrobacter winogradskyi* to source-separated feces, which boosts the fertility by optimizing the nitrogen composition as well as removing heterotrophic microorganisms [17,18]. However, it is essential to check if this method is profitable for hygienic purposes by investigating the effect of the aforementioned nitrifying microorganisms on the fate of fecal indicators.

Therefore, the two objectives of this study were: (1) To investigate the fate of intestinal microorganisms in source-separated feces by tracking *Escherichia coli* and total coliforms as fecal indicators, and (2) to examine the effect of adding nitrifying microorganisms on this fate.

## 2. Materials and Methods

### 2.1. Sample Preparation

The sampling procedure was previously described [18]. Fresh samples (collected within 24 h) of raw source-separated feces were obtained from the feces storage tank of a ROS system using a 20-L sterile sampling bag, as previously described [2]. The construction and operation of the ROS system were carried out by the GnV Company (Gunpo, Korea). This system is located in a public park near the West Suwon Lake Prugio Residential Complex Phase 1 at Gunpo City, Gyeonggi Province, Republic of Korea (37°18′00″ N, 126°47′11″ E). The same company provided two stabilized bio-seed solutions. One solution contained 6 × 10^6^ *N. europaea* cells per 100 mL and the other solution contained 8 × 10^5^ *N. winogradskyi* cells per 100 mL. Both bio-seed solutions contained various microbial growth promoters including amino acids, vitamins, and minerals.

Twenty-one sets of samples were prepared in 1-L beakers as a batch system, with each set containing three samples (total of 63 samples), which enabled replication of the experiment. Each sample included 300 g of feces, which represents the usual defecation amount of one event [8]. One set of samples was left intact as the control, whereas varying amounts of bio-seed were added to the other sets of samples. By diluting the bio-seed solutions, a range of 1000–20,000 cells of *N. europaea* bio-seed were added per 1 g of feces, in 1000-cell increments. In addition, a specific constant amount of 10,000 *N. winogradskyi* cells per 1 g of feces were added to the samples of each set, except for the control set.

Using laboratory mixers (model EW-04555-00; Cole-Parmer, Vernon Hills, IL, USA), the samples were mixed at 45 rpm. After mixing, the samples became homogeneous, soft, and creamy. During the experiment, the samples were turned over twice daily using the mixers at a speed of 45 rpm for 10 min. To avoid bacterial contamination, all beakers and mixing blades were first disinfected using 70% ethanol provided by Samchun Chemical (Pyeongtaek, Korea) and were subsequently placed in an ultraviolet (UV) sterilizing oven (Model SW305H; Shimwon, Incheon, Korea) using four 15-W UV lamps for 90 min.

During the experiment, all the sets of samples were maintained under similar conditions. The initial temperature of the samples and the experimental environment were maintained at 25 °C, the optimum temperature at which the nitrifying bio-seeds are active [19]. A soil pH meter (model AB927; Lee Valley Tools Ltd., Ogdensburg, NY, USA) was used for pH measurement.

### 2.2. Analysis of Characteristics 

Every day, using 1-g samples of each set, liquid extracts of feces were prepared with 100 mL distilled water. The water samples were placed in the UV sterilizing oven for 12 h before the experiment to avoid any unwanted bacterial interaction. These extracts were used to measure *E. coli* and total coliforms. For this purpose, 3M™ Petrifilm™ *E. coli* and Total Coliform Count Plates (3M, St. Paul, MN, USA) were used. Results were interpreted following ISO 6222 [20]. The detection limit for this method was two log_10_ CFU/g feces.

Using three samples in each set, all measurements were performed in triplicate and an arithmetic mean was calculated. Standard errors are used to provide error bars. The significance of changes in fecal indicators was assessed by Student’s *t*-test analyses (α = 0.05). Furthermore, one-way statistical analysis of variance (ANOVA) was used to evaluate the differences between the trends of removal of fecal indicators achieved in treatment with the different initial addition of bio-seeds. Each dependent parameter, e.g., microorganism population and pH, is strongly related to two independent ones, comprising the time and the initial addition of bio-seeds. Therefore, to present such a bivariate model, the results were plotted as contour diagrams through local polynomial regression [18].

Table 1 presents the initial characteristics of the intact feces sample. The experiments lasted for 30 days. However, the condition of all the samples stabilized after approximately 20 days, with no significant changes detected thereafter. 

## 3. Results

### 3.1. Fate of E. coli and Total Coliforms in Intact Feces

**Figure 1:**
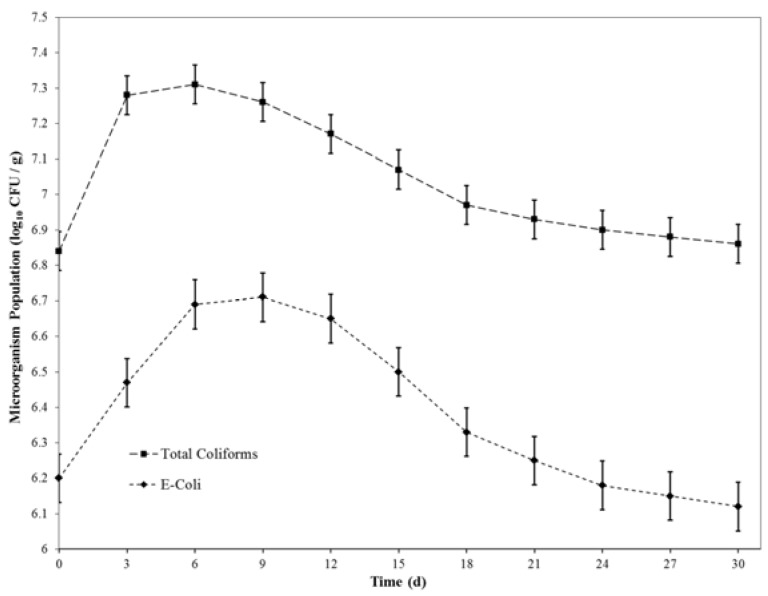
Changes in the microorganism population in 1 g of intact feces with time.

Figure 1 presents the changes in the numbers of *E. coli* and total coliforms in intact feces. For a short period initially, the microorganism population increased significantly (*p* < 0.001). However, after this initial growth phase, the microbial population eventually decreased. After 21 days, there was no significant change in the microbial population (*p* > 0.06), implying that the rates of microbial growth and death may have become equal.

### 3.2. Effect of Adding Nitrifying Microorganisms on the Fate of E. coli and Total Coliforms

**Figure 2:**
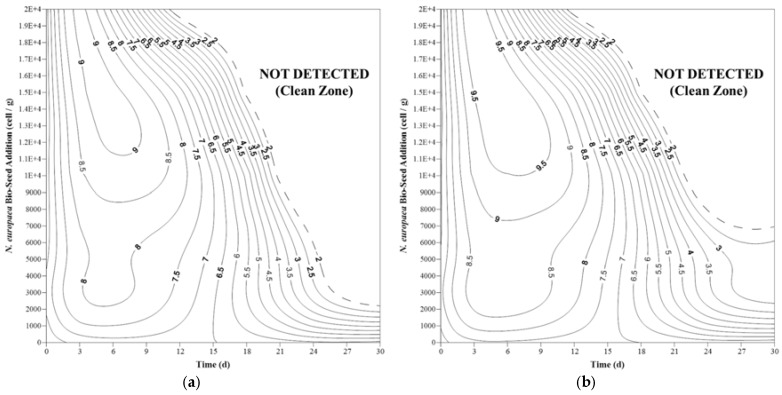
Changes in (**a**) total *E. coli* population and (**b**) total coliform population in experimental samples with different amounts of nitrifying bio-seeds per 1 g of feces; numbers in contours represent log_10_ CFU/g.

Figure 2 presents the changes in the population of *E. coli* and total coliforms by adding different amounts of *N. europaea* bio-seed along with a constant amount of *N. winogradskyi* bio-seed. The general trend observed after adding varying amounts of nitrifying microorganisms on *E. coli* and total coliforms was not statistically different (*p* > 0.03).

The addition of bio-seeds initially increased the microbial population, which decreased over time. The minimum amounts of *N. europaea* bio-seed required to achieve the clean zone were 3000 and 7000 cells per 1 g feces, respectively, for *E. coli* and total coliforms. For both *E. coli* and total coliforms, the highest microbial population was observed when the amount of *N. europaea* bio-seed added was higher than the constant amount of *N. winogradskyi* bio-seed (10,000 cells to 1 g feces).

**Figure 3:**
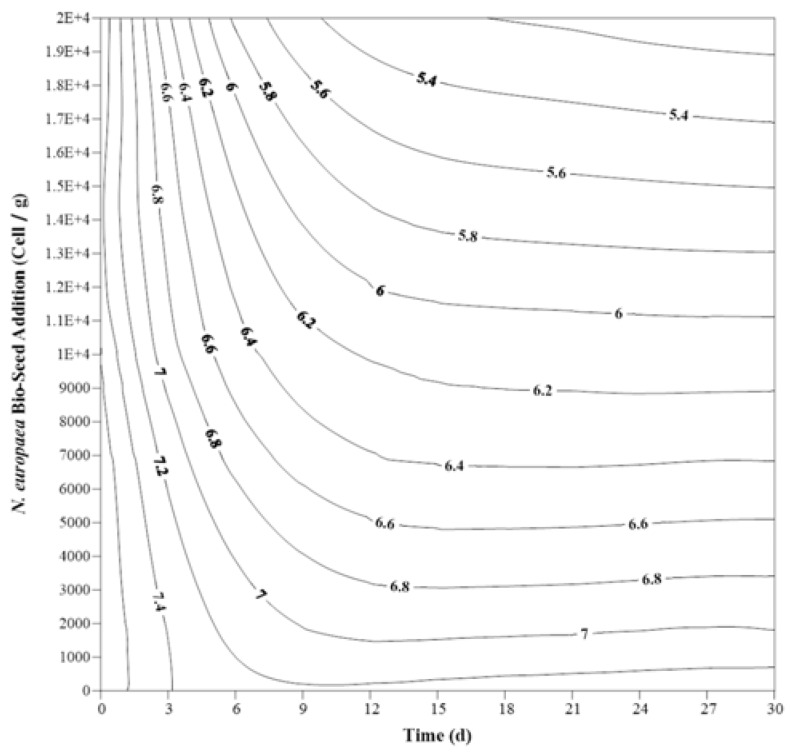
Changes in pH in experimental samples with different amounts of nitrifying bio-seeds in 1 g of feces; numbers in contours represent pH.

Significant reduction in pH was observed (Figure 3), as was also reported previously [18]. This trend was more rapid with higher addition of *N. europaea* bio-seed, and probably because of the nitrification process.

### 3.3. Comparison of Time Required for Removal of E. coli and Total Coliforms

**Figure 4:**
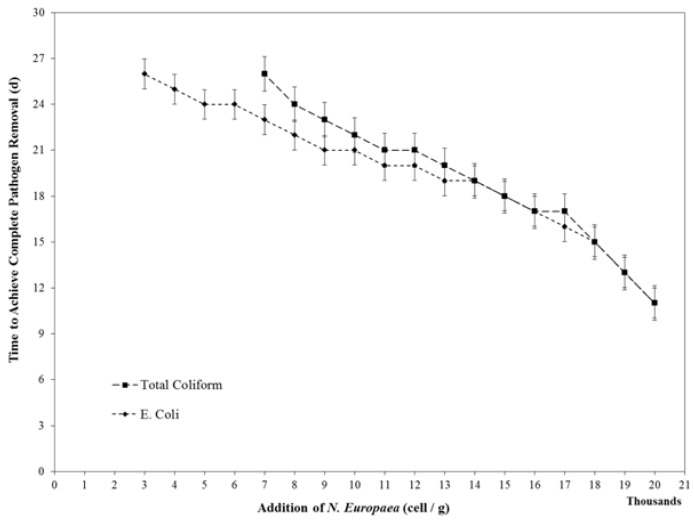
Changes in pH in experimental samples with different amounts of nitrifying bio-seeds in 1 g of feces; numbers in contours represent pH.

Figure 4 presents the data of the time required for the complete removal of *E. coli* and total coliforms following the addition of different amounts of nitrifying microorganisms. With low addition of nitrifying microorganisms of 0–3000 cells and 0–7000 cells to 1 g feces, there was incomplete removal of *E. coli* and total coliforms, respectively. Consistently, adding a higher amount of nitrifying bio-seeds reduced the time required for complete removal of *E. coli* and total coliforms.

As mentioned previously, the minimum amount of bio-seeds required for complete removal of *E. coli* was less than that for total coliforms. However, as the amount of nitrifying bio-seeds added increased, the time required to achieve a clean zone for total coliforms approached that of *E. coli*. For instance, in the case of adding 20,000 cells per 1 g feces, the time required to achieve clean zones for *E. coli* and total coliforms was the same (11 days).

## 4. Discussion

There are two important phases in the trends of the fate of *E. coli* and total coliforms: The growth phase followed by the reduction phase. The addition of nitrifying microorganisms enhances both phases. The enhancement observed in the growth phase might be due to the reduction of ammonia as a result of nitrification, as the presence of ammonia limits the growth of *E. coli* and some other coliforms [18,21].

It has also been demonstrated that the addition of nitrifying microorganisms can significantly enhance the degradation of source-separated feces by improving the growth of heterotrophic microorganisms, leading to higher consumption of available organic carbon compounds [18]. Since the availability of organic compounds plays a vital role in the vitality of microorganisms, the number of fecal indicators declines significantly after the rapid degradation of Total Organic Carbon (TOC) [18].

The enhancement in the reduction phase may also be explained by considering the reduction of pH. As the nitrification process continues with time, the production rate of nitrate increases, especially with the addition of more bio-seeds. In this case, the pH drops rapidly which makes the condition unfavorable for habitat microorganisms, leading to the enhancement in the reduction phase [18,22]. However, this cannot be considered as a conclusive reason, since the pH stress response of *E. coli* is sophisticated, robust, and versatile, and since other studies have described the acid habituation behavior of *E. coli* [14,23].

There are concerns about the hygiene of ROS systems. Addressing these concerns with sustainable solutions can help make these sanitation systems more acceptable. This study suggests an innovative feces treatment method through the addition of nitrifying microorganisms.

In onsite treatment processes in ROS, it is essential to apply the optimal amount of bio-seed to the source-separated feces. Hashemi and Han suggested that an additional amount of 7000–8000 *N. europaea* cells to 1 g feces, along with 10,000 *N. winogradskyi* cells per 1 g feces, can probably be considered optimal, because under this circumstance, a sustainable 1:1 ratio of nitrate to ammonium and a pH of between 6.2 and 6.4 was achieved, meeting the criteria for standard fertilizer [18]. The results of the current study show that under such a situation it takes about 23 and 26 days to achieve total removal of *E. coli* and total coliforms, respectively. Using a higher initial addition of *N. europaea* bio-seed can reduce the required time for the treatment, which can be useful in a circumstance of high feces input.

Consequently, fecal storage and reactors in ROS systems must be designed reasonably. The mixture of fresh and stored feces should be avoided, as the fresh feces contain pathogens and will reduce the efficiency of this treatment method. It is proposed that the time interval should be defined for treating the gathered feces and preparing them for utilization. Using smart systems supported by information technology can be useful for measuring the exact amount of feces input, determining the optimum initial addition of bio-seeds, and provide it by the proper dilution process. In addition, the enhancement in the growth phase caused by the addition of nitrifying microorganisms is useful in boosting the degradation process of the source-separated feces [18]. Therefore, it is essential to limit access to the treatment reactors until the reduction phase starts.

## 5. Conclusions

In this study, we investigated the fate of *E. coli* and total coliforms in stored feces. The reduction rate of microorganisms in these samples was slow. This poses potential health challenges in the case of a lack of treatment or improper sanitation practices, such as open defecation. The addition of nitrifying microorganisms proved useful for reducing the viable numbers of *E. coli* and total coliforms in feces. This can be useful for ROS systems that are unable to perform proper composting or other appropriate treatment processes for feces, especially in low to middle-income countries. An innovative approach for designing fecal treatment reactors is thus an essential future direction and deserves to be investigated in separate research.

## Figures and Tables

**Table 1:** Initial characteristics of intact feces.

Parameters	Average	Standard Deviation
pH	7.8	0.22
log_10_ CFU *E. coli*/g	6.2	0.12
log_10_ CFU Total Coliforms/g	6.9	0.10
